# Causes of volcanic unrest at Mt. Spurr in 2004–2005 inferred from repeated tomography

**DOI:** 10.1038/s41598-018-35453-w

**Published:** 2018-11-30

**Authors:** I. Koulakov, S. Z. Smirnov, V. Gladkov, E. Kasatkina, M. West, S. El Khrepy, N. Al-Arifi

**Affiliations:** 1 Trofimuk Institute of Petroleum Geology and Geophysics, SB RAS, Prospekt Koptyuga, 3, Novosibirsk, 630090 Russia; 2 Novosibirsk State University, Novosibirsk, Russia, Pirogova 2, Novosibirsk, 630090 Russia; 3 Sobolev Institute of Geology and Mineralogy SB RAS, Prospekt Koptyuga, 3, Novosibirsk, 630090 Russia; 4 Geophysical Institute, University of Alaska Fairbanks, Fairbanks, Alaska USA; 5 King Saud University, Riyadh, Saudi Arabia, P.O. Box 2455, Riyadh, 11451 Saudi Arabia; 6 National Research Institute of Astronomy and Geophysics, Seismology Department, NRIAG, Helwan, 11421 Egypt

## Abstract

Mt. Spurr is the largest active volcano in Alaska of high explosive potential. The most recent activity, including two recent magmatic eruptions in 1953 and 1992, has occurred via the flanking Crater Peak. From 2004 to 2006, strong seismicity, gas flux, and heating were observed in the summit area, which had remained inactive for more than 5 Ka. To understand the cause of this reactivation, we performed repeated tomography inversions that clearly imaged the magma reservoir beneath Mt. Spurr and showed temporal changes in its shape and intensity. During the two years preceding the unrest, we observed ascension of the upper limit of the reservoir-related anomaly from a depth of 5 to 3 km below the surface, accompanied by strong seismicity. During the following years, the shape of the anomaly remained unchanged, but its intensity weakened. These observations may indicate the release of fluids from the ductile reservoir and fast upward ascent through the brittle cover that caused intensive seismicity and gas flux during the unrest from 2004 to 2006. The origin of this zone will possibly cause a resumption of explosive eruptions in the summit area of Mt. Spurr.

## Introduction

Active volcanoes are dynamic geological structures in which considerable changes in physical properties may occur very quickly^1,2^. Among the main causes of such changes is the migration of fluids, phase transitions, and degassing within the volcanic plumbing system that are the main reasons for explosive eruptions^3,4^. Investigating these changes provides an important key to understanding the processes of preparation and realization of volcanic eruptions.

**Figure 1:**
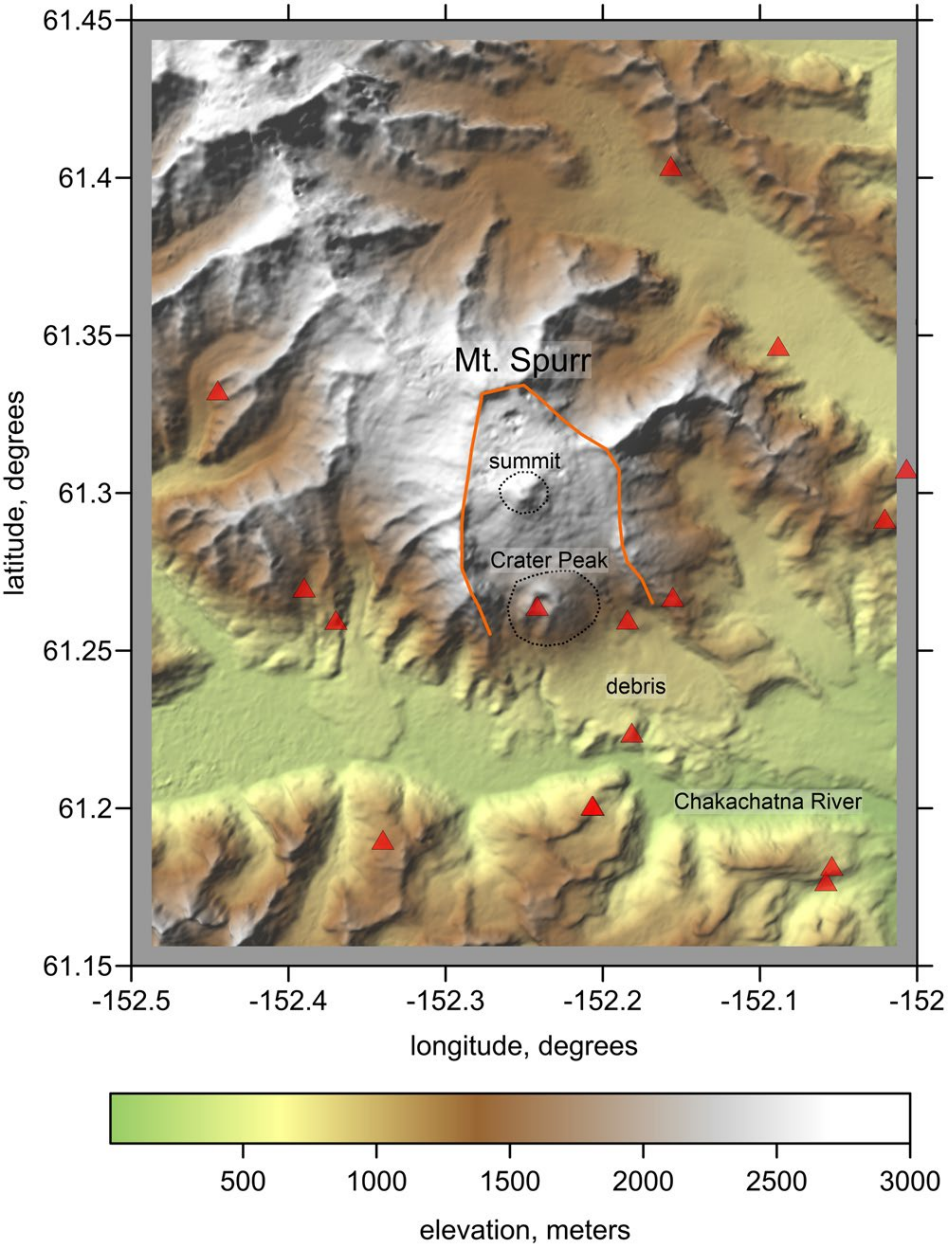
Topography of the study area (extracted from Global Multi-Resolution Topography, GMRT)^28^ and distribution of the seismic stations (red triangles). The limits of the horseshoe caldera are highlighted by the orange line. The summit of Mt. Spurr and Crater Peak are indicated by the dotted line.

Among several examples of dynamic volcanic systems is Mt. Spurr, which is the highest and easternmost active stratovolcano in the Aleutian Arc of Alaska at an elevation of 3,374 m (Fig. 1). The summit of Mt. Spurr is a large dominantly andesitic lava dome inside a 5-km diameter horseshoe-shaped caldera, which originated from a large explosive eruption that occurred approximately 10–12 thousand years ago^5^. The recorded traces of a huge debris avalanche from this event, with deposited blocks reaching a diameter of 100 m, demonstrates the strong explosive potential of this volcano which may have a global impact if such an eruption were to occur today.

Prior to the unrest from 2004 to 2006, the summit area of Mt. Spurr was considered inactive. The latest summit eruption had been dated at ~5.2 Ka^6^. Most Holocene activity on Spurr had been concentrated at the younger satellite vent, Crater Peak, 3.5 km south of the main summit. During the 20^th^ century, Crater Peak hosted two recent explosive eruptions in 1953^7^ and 1992^8^. Both of these eruptions produced pyroclastic flows and deposited ash on the city of Anchorage and other populated areas at a distance of 130 km^9^.

**Figure 2:**
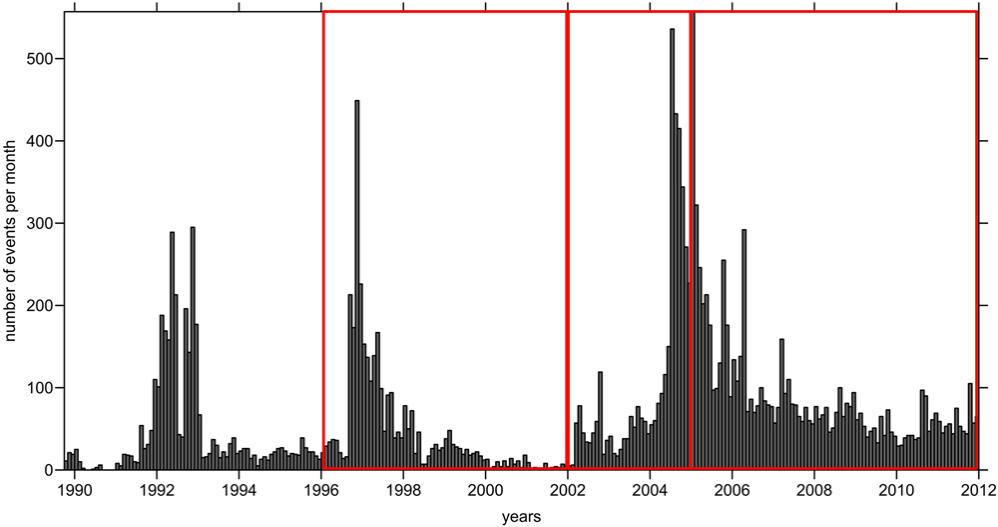
Histogram of the seismic events beneath Mt. Spurr from 1990 to 2012. The red line highlights the three time periods considered in this study.

During 2002, evidence of awakening occurred in the long-dormant summit area of Mt. Spurr. For the two following years, increasing seismicity was observed beneath the summit (Fig. 2) culminating in degassing at the summit^10^. The 2004–2005 seismic swarm included both volcano-tectonic earthquakes beneath the summit at a depth of 5–10 km below sea level (bsl), and deep long-period earthquakes^10,11^. The maximum number of events (more than 100 per week) was observed from July to November 2004 and in April 2005 (Fig. 2). Then, during the period from July 2005 to March 2006, seismic activity gradually decreased to 20–30 events per week^12^. An exception was a swarm from April 11 to 12, 2005, when 156 volcano-tectonic events at ~5 km bsl were detected^11^. Elevated heat flow and gas flux during 2004–2005 drastically changed the morphology of the ice cover^12^. The area of the ice cauldron in the crater reached 80,000 m^2^ in July 2005 and then remained unchanged until the end of the activity in 2006. According to Doukas and McGee^13^, the flux of CO_2_ reached 1400 tons per day in 2004, and then gradually decreased to 200–400 tons per day in 2006. In contrast, the flux of SO_2_ was nearly absent during the beginning of activity and then increased to 400 tons per day in 2006. The amount of water steam, which was not directly measured, was approximately 10 times greater than that of the CO_2_ typical in these types of volcanoes^14^. Thus, the total gas flux may have reached 10–15 thousand tons per day. Based on interferometric synthetic aperture radar (InSAR) observations, Lu and Dzurisin^15^ calculated a total inflation of ~5 cm in the Mt Spurr area during the unrest period of 2004–2006. They estimated that the source of this deformation was an expansion in volume of 0.05–0.06 km^3^ at 10–14 km depth. All of these observations resulted in a high hazard level until early 2006, but no magmatic eruption occurred. Fumarolic activity in the crater was observed during the winter of 2011–2012.

The unrest of Mt Spurr during 2004–2006 was monitored by a deployed temporary seismic network in addition to permanent stations in the area. Recorded seismicity and arrival times of the P and S seismic waves were used to build the tomography model of the crust beneath Mt. Spurr^16^. This model showed two finger-shaped anomalies of high Vp/Vs ratio at a distance of ~8 km from one another. One of the anomalies reached a depth of ~4 km below the surface in the area where most of the geothermal activity and gas emission occurred during 2004–2005. The top of the second anomaly was at a depth of 20–25 km beneath Crater Peak. Note that this tomography model appeared significantly different from the earlier P-wave velocity model of Power *et al*.^17^. The reason for this difference might have been the significantly larger data amount in the latter case, but it could also have been because of changes in the magma system.

The temporary network deployed during the unrest during 2004–2005 allowed for the development of a high-resolution tomography model. However, because of the short term of the deployment of the portable stations, these data could not provide information regarding temporal changes in the plumbing system beneath the volcano. In the present study, we used data of permanent stations of uniform quality and spatial coverage over decades that provided a possibility to detect four-dimensional (4D) variations in seismic velocities and associate them with the volcanic activity of Mt. Spurr.

## Methods

Studying temporal changes in seismic velocities using local earthquake tomography is a challenging task. Several attempts have been made to study time-dependent seismic structures in volcanoes and other tectonically active structures^1,2,18^. The major problem in most of these studies is that passive non-controlled sources typically provide different ray coverage in different time episodes, and this may cause much stronger phantom velocity changes than the actual temporal variations in the seismic properties at depth. Therefore, the results of such quasi-4D tomography studies should be considered with prudence and interpreted only if the changes are very strong. For the very small changes that occur during most geological processes, repeated tomography inversions are not capable of distinguishing real variations from artifacts related to changes in data coverage. Among the alternative solutions is using ambient noise correlation^19–21^. However, for small-aperture seismic networks installed on active volcanoes, the noise correlation may be strongly affected by changes in dominant noise directions. In addition, noise-based methods in volcano-scale studies typically provide information regarding shallow structures a few kilometers below the surface that might appear to be useless for studying deep-seated magma reservoirs^19^.

Here, we used an approach recently developed and tested in different structures, such as the area of the Tohoku-Oki earthquake^22^ and at the Nevado del Ruiz volcano^23^. This method is based on selection of dataset series with similar distributions of events and seismic rays. The tomography inversion results calculated for such data largely represent the actual changes in seismic properties. Some artifacts still occur because of non-perfect similarity in the datasets that can be easily estimated using synthetic tests. In the case of the Nevado del Ruiz volcano, this method provided gradual changes within a prominent Vp/Vs anomaly beneath the crater that was interpreted as deflation of the fluid-saturated magma reservoir during degassing episodes.

We implemented a similar approach to study the changes beneath Mt. Spurr. Data of the permanent seismic network were grouped in two series of time periods (Fig. 2). In the first series, we analyzed the data from 1996 to 2001 and from 2002 to 2004. In the second case, we considered the data from 2002 to 2004 and 2004 to 2012. For each series, during both time periods, we found events at a distance of less than 0.5 km from one another, and for these, we selected picks recorded by identical stations and of the same phase (P or S). The data distributions in all series are shown in Supplementary Fig. S1. Further detail regarding the data and the inversion procedure is described in the Supplementary Information.

## Results and Discussion

**Figure 3:**
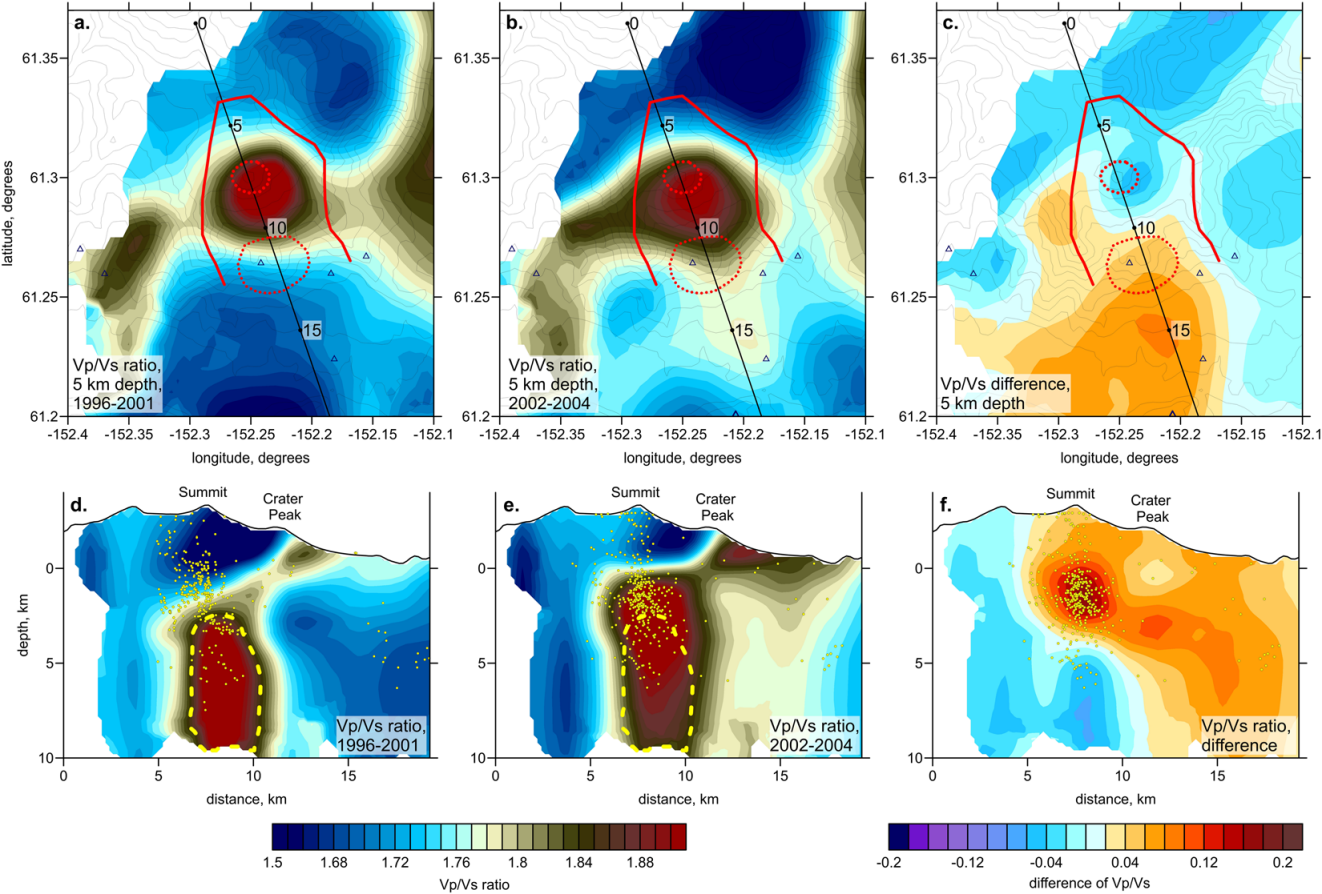
Distribution of the Vp/Vs ratio derived from the inversion of datasets from two time periods: (1996–2001) and (2002–2004). The results are plotted at 5 km depth (upper row) and in the vertical section (lower row). The location of the section is indicated in the maps. The right column presents the difference between the Vp/Vs distributions in the corresponding horizontal and vertical sections. The yellow dots in the vertical sections depict the events within a distance of 5 km from the profile. The yellow dashed line highlights the contour line of Vp/Vs = 1.85 corresponding to the data from 1996 to 2001. The red solid line depicts the limits of the caldera; the red dotted lines indicate the Mt. Spurr summit and Crater Peak.

**Figure 4:**
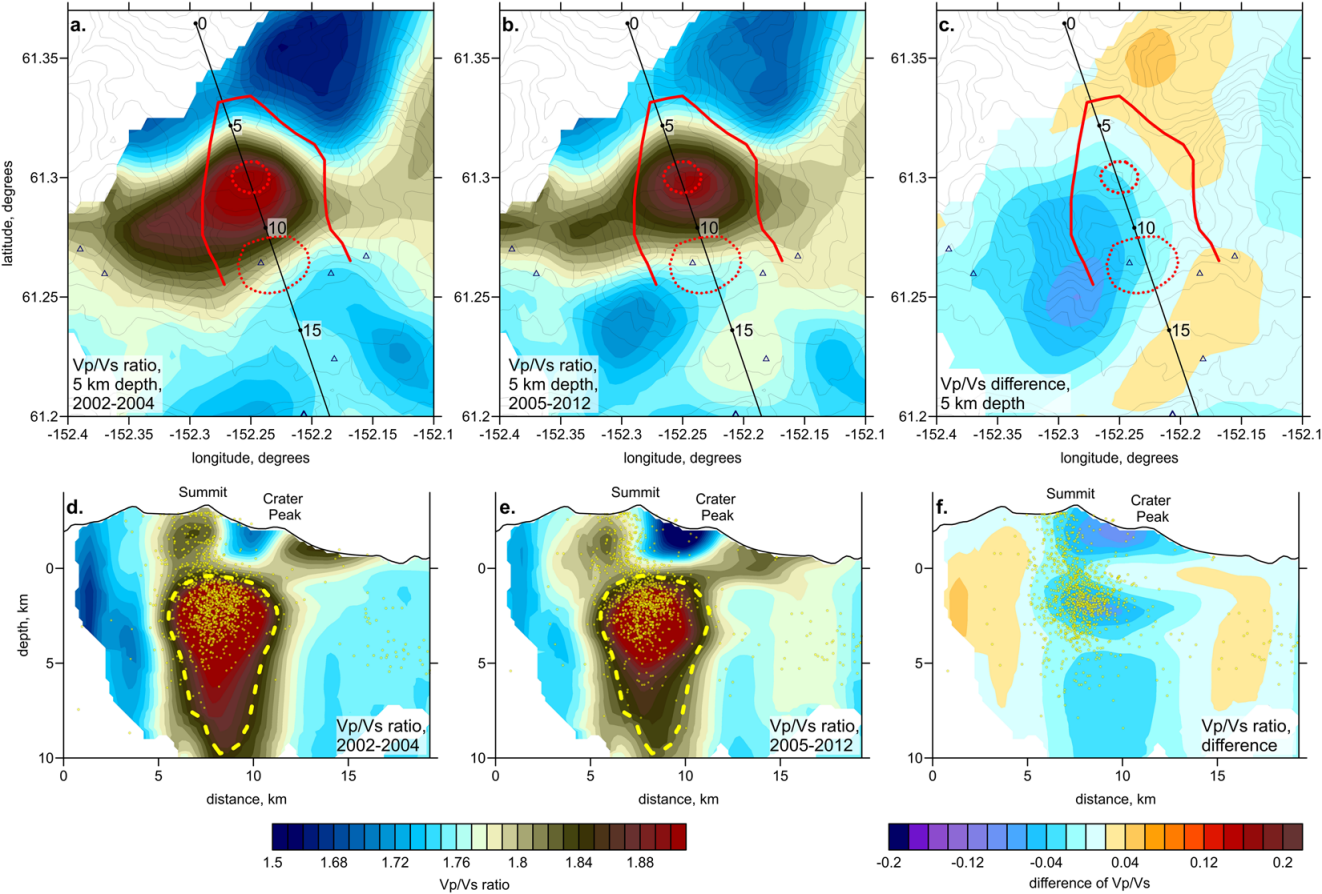
Same as in Fig. 3, but for the time periods of 2002–2004 and 2005–2012.

The results of the inversions for the two series of datasets are shown in Figs 3 and 4. Here, we present only the distributions of the Vp/Vs ratio in one vertical and one horizontal section, as well as the difference between the models for each series. The corresponding distributions of the P- and S-wave velocity anomalies are shown in the Supplementary Information in Figs S2 and S3. During all time episodes, one can observe one prominent columnar anomaly of a high Vp/Vs ratio beneath the summit area, likely associated with the magma reservoir. Within this anomaly, the ratio reaches a value of 2, whereas in the surrounding areas, the Vp/Vs is generally low (to 1.5). Another common feature observed during all periods is an inclined anomaly connecting the reservoir-related anomaly to Crater Peak, where most of the recent magmatic eruptions have occurred.

The models derived in this study are generally consistent with the previous higher-resolution tomography model of Koulakov *et al*.^16^ based on data from the temporary stations deployed during the 2004–2005 unrest period. The columnar anomaly with the high Vp/Vs ratio beneath the main summit of Mt. Spurr corresponds to the shallower “finger” interpreted by Koulakov *et al*.^16^ as a volatile conduit. The deeper conduit, identified in the previous study, is not detected here because of insufficient data coverage. There is less correspondence with the earlier tomography study of Power *et al*.^17^, but their seismic model is not in contradiction to the results obtained in this study. For example, in both cases, a high Vp is observed beneath the summit area and a lower Vp beneath Crater Peak. Based on this observation, Power *et al*.^17^, who did not use S-waves, concluded that the main conduit is beneath Crater Peak where the P-wave velocity is low. This differs from our interpretation, which is mainly based on the distributions of Vs and the Vp/Vs ratio.

The results of inversions for the first series corresponding to the periods of 1996–2001 and 2002–2004 are shown in Fig. 3. Comparing the models derived for the different time periods, there are some important differences. The yellow contour line in the vertical section shown in Fig. 3 indicates a level of Vp/Vs = 1.85 corresponding to the period 1996–2001. Relative to this contour, the anomaly during the second period of 2002–2004 appears to have shifted upward to approximately 2 km. The plot with the difference shown in Fig. 3f shows a strong increase in Vp/Vs at a depth interval of 0–2 km (with respect to sea level). At deeper levels, the Vp/Vs ratio seems to decrease.

The results for the 2^nd^ series of 2002–2004 and 2005–2012 shown in Fig. 4 appears more similar compared to the previous case shown in Fig. 3. Here, the shape of the central anomaly of the high Vp/Vs ratio remains unchanged, but one can only detect some decrease in its amplitude. The synthetic modeling results shown in Supplementary Figs S4 and S5 show that the velocity changes observed in both series are robust and not related to variable ray coverage.

Note that the results for the same period of 2002–2004 corresponding to the two series shown in Figs 3a,d and 4a,d appear slightly different, although they should represent the same structures. This difference is merely caused by different data coverage. Larger number of events in one case requires defining different set of controlling parameters than in another case, which affects the tomography results. This result illustrates how difficult the interpretation of repeated tomography may be if a difference in ray coverage is not considered.

Based on the obtained results, we propose a possible scenario of the Mt. Spurr volcanic activity. We use the general concept of the model developed by Fournier^24^, who described hydrothermal processes related to fluid migration between the magma reservoir and surface. This model presumes the location of the magma source at 3 km below the surface, similar to our case. This concept was already used by Mercier and Lowell^25^ to describe the nature of the “missed eruption” of Mt. Spurr. However, they used the tomographic model of Power *et al*.^17^, in which only P-wave velocities were available. Our results provide important additional information that allows for further development of the volcano feeding model.

**Figure 5:**
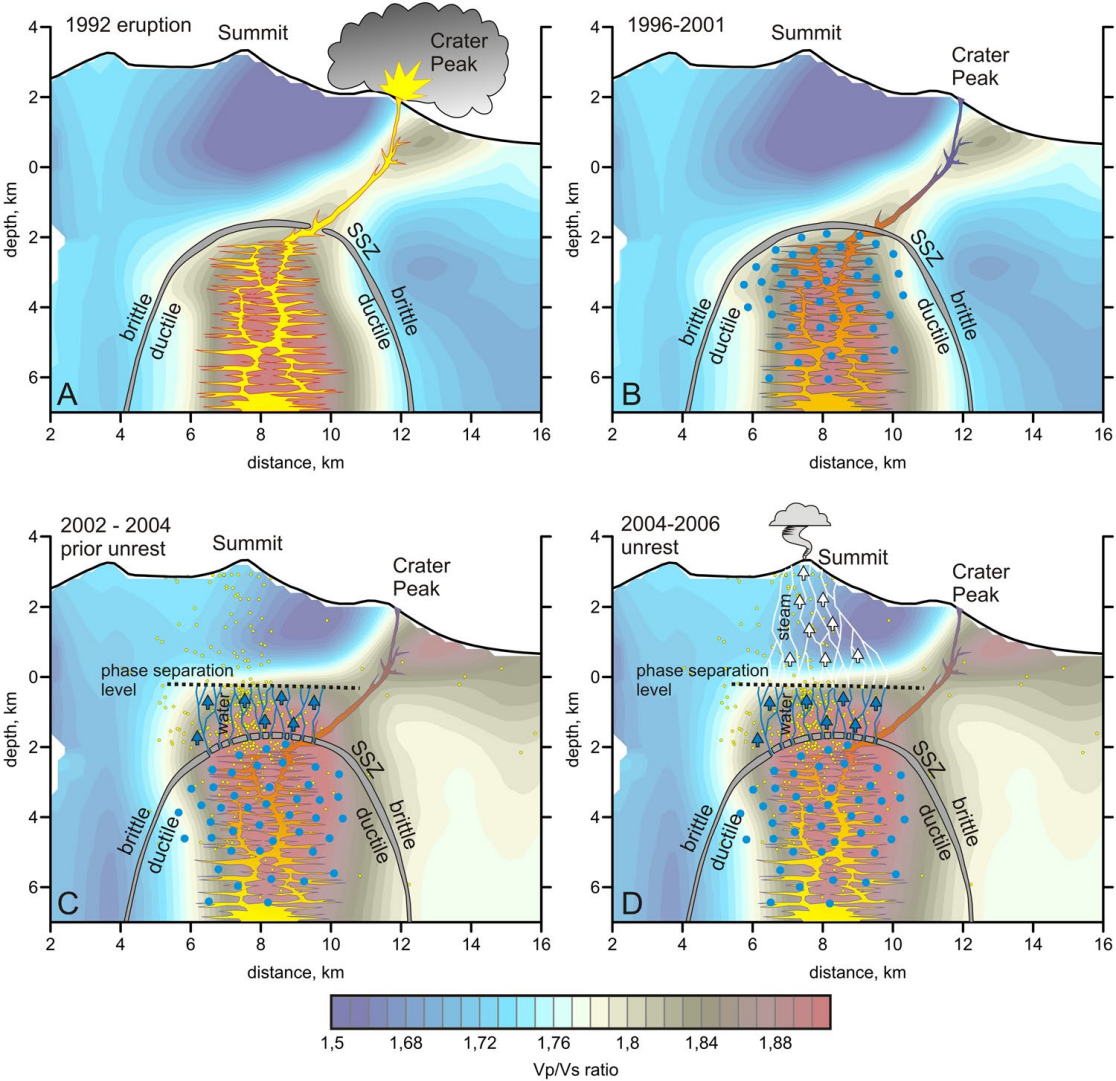
Conceptual scheme of the possible stages of the Mt. Spurr volcanic activity. (**A**) Magmatic eruption in 1992 via the flank conduit. No activity occurred in the summit area, which was screened by a non-permeable self-sealed zone (SSL) depicted by the gray layer. (**B**) Relaxation period from 1996 to 2001: the flank conduit becomes impassable. Fluids (blue dots) accumulate in the ductile part of the magma reservoir. (**C**) Beginning of the unrest from 2002 to 2004; the strong seismic activity without apparent degassing on the surface represents disruption of the SSL and penetration of liquid fluids into the brittle part of the crust (blue arrows and lines). (**D**) Volcanic unrest from 2004 to 2006; liquid fluids reach the phase separation level and then continue ascending as gas (white arrows and lines).

The prominent high-Vp/Vs ratio body observed in our tomography models beneath the summit area likely represents the shallowest magma reservoir, which is directly responsible for the recent volcanic activity. In all models, this body seems to be connected to the flanking Crater Peak via an inclined high-Vp/Vs anomaly representing the conduit that fed the recent eruptions of Mt. Spurr. Fig. 5A shows a schematic of the magmatic explosive eruption that occurred at Crater Peak in 1992. Unfortunately, we did not have sufficient data to build the model for the period of the 1992 eruption; however, based on the general similarity of all the tomography results in this study, we propose that the same magma reservoir and conduit existed in 1992. We believe that excessive pressure within the magma reservoir caused opening of this conduit bringing t molten magmatic material and volatiles to the surface.

The first time period (1996–2001) considered in our study corresponds to the relaxation stage between the two volcanic unrest periods (Fig. 5B). Cooling of the magma in the conduit to the flank crater over nearly 10 years resulted in it becoming impermeable. We believe that during this time, a number of volatiles arrived from a deeper magma reservoir (such as that at 10–12 km depth presumed from the observed ground deformation) via existing conduits of liquid magma. In ductile material, migratory fluids harden, and gradually accumulate in the upper part of the reservoir the (blue circles in Fig. 5). During this stage, passing of the volatiles to the brittle part of the crust was prevented by a rigid layer, termed a self-sealed zone (SSZ)^25^, as shown in Fig. 5 by the gray color.

A new seismic unrest started in 2002. We propose that during this stage, the amount of the accumulated fluids in the ductile part reached a critical value and/or their temperature became too high. As shown in Fig. 5C, these fluids could have destroyed the SSZ that screened the magma reservoir from the brittle upper crust as discussed in Fournier *et al*.^24^. In contrast to the ductile material, in brittle rocks, a system of fractures can easily form, which is favorable for rapid migration of fluids. Thus, the seismicity between 2002 and 2004 was probably caused by fracturing of the brittle layer between a depth of 2 km and sea level. The filling of cracks in this layer with liquid fluids changed the Vp/Vs ratio from low to high.

Figure 5D shows the final stage of the volcanic unrest corresponding to the period from 2004 to 2006. At a zero level (3 km below the surface), we can observe the transition from a high- to a low-Vp/Vs ratio. The same transitions at similar depths have been observed in other strongly degassing volcanoes, such as Nevado del Ruiz^23^ and Gorely in Kamchatka^26^. This limit might represent the level of phase separation in which the liquid phase is converted to gas. It is known that the Vp/Vs ratio is a sensitive indicator allowing for the distinguishing of gas- and liquid-saturated rocks. A high Vp/Vs is associated with the presence of liquids (melts and fluids), whereas a low Vp/Vs ratio may indicate the presence of gas^27^. In this case, the magmatic volatiles are converted to gas ~3 km below the surface and then migrate upward to the summit area as shown by the white arrows in Fig. 5D. This scheme is consistent with the geothermal model of Mercier and Lower^25^, in which the “boiling hydrothermal layer” is also defined at sea level and at approximately 3 km below the surface. Upward migration of steam, as well as CO_2_, SO_2_, H_2_S, and other volcanic gases, caused the strong heating and intensive fumarolic activity observed in the summit area from 2004 to 2006. As shown in Fig. 4, the shape of the magma-associated anomaly did not considerably change from 2005 to 2012 after the surface manifestations of the volcanic activity. We only detected weakening of the anomaly intensity that may have indicated that because of the intensive output of fluids, the magma had lost some amount of dissolved volatiles.

## Conclusion

In summary, we can conclude that in the case of the selection of datasets of similar configurations, repeated local earthquake seismic tomography is able to show temporal variations in underground seismic velocities. These variations are particularly clear beneath volcanoes in which active processes in the plumbing system may change the structure very rapidly compared to that of other geological structures. We propose that the major role of such changes is the migration of fluids, phase transitions, and other related processes.

With regard to Mt. Spurr, we detected considerable changes in seismic velocities that allow for the tracking of the evolution of the plumbing system during the unrest phases. The upward shift of the upper limit of the magma reservoir-related anomaly during the first stage of the unrest from 2002 to 2004 may indicates the release of fluids and their ascent to ~2 km depth. We propose that, previously, the summit area was screened from the magma reservoir by a rigid layer that prevented penetration of fluids and melts. From 2002 to 2004, this screen was destroyed by hot fluids, affecting the summit area. Although the unrest from 2004 to 2006 did not result in a magmatic eruption, it appears to be very important for the future evolution of Mt. Spurr. It is possible that demolition of the rigid cover has led to the creation of a weakened zone beneath the summit that may be transformed into a magmatic conduit. Therefore, we cannot exclude the possibility of a resumption of magmatic eruptions in the summit area of Mt Spurr after more than 5,000 years of inactivity.

## Electronic supplementary material

Supplementary information
