# Supplementary material for: Causes of volcanic unrest at Mt. Spurr in 2004–2005 inferred from repeated tomography
Source: Sci Rep. 2018 Nov 30;8:17482. doi: 10.1038/s41598-018-35453-w (PMC6269431; doi:10.1038/s41598-018-35453-w)
Supplement: Supplementary file 1 — Supplementary information [file 41598_2018_35453_MOESM1_ESM.pdf]

Causes of volcanic unrest at Mt. Spurr in 2004-2005 inferred from repeated tomography

I. Koulakov [1,2*], S.Z. Smirnov [3], V. Gladkov[1], E. Kasatkina[1], M. West[4], S. El Khrepy[5,6], N. Al-Arifi[5]

Supplementary Information

This material includes the description of data and algorithms. It also presents the results of the inversion of experimental data and synthetic tests.

Data and algorithms for repeated tomography

In this study, we used catalogue data of the permanent station network deployed on Mt. Spurr (Figure 1 of the main paper and Table S1). In addition to the local network, we used several closest regional stations that recorded strongest events beneath Mt. Spurr. In total, twenty-six stations were involved in recording the local seismicity. However, during selected periods, the number of actual stations ranged from 11 to 16. There might be a concern that during the long observation period, varied instrumentation characteristics might cause bias of picking time in some periods. A possibility of regular errors related to this factor was explored, for example, for the data of the ISC Bulletin[29]. However, as seen in Table S1, during the considered period, the instrumentation and the picking technique did not change considerably that enables more or less constant quality of data in the catalogue. The initial catalog includes 18,148 events from 1989 to 2012. The total number of picks was 143,455 and 101,951 for the P- and S-waves, respectively.

Details of the algorithms of repeated tomography including the data selection are described in Vargas et al.[23]. For this study, we selected three time intervals: 1996–2001 (1), 2002–2004 (2), and 2005–2012 (3), as shown in Figure 2 of the main paper. This selection was done regarding the different stages of volcano activity, but also considering the distribution of events such that the following procedures provided sufficient data for tomography. We performed repeated tomography inversions for two pairs of datasets, namely (1) –(2) and (2) –(3). We selected pairs of events that occurred in each of the two datasets and at distances less than 0.5 km. For these, we considered the picks recorded by the same stations and having the same type of waves, P or S. Among all the combinations of paired events, we selected those having the maximum number of common phases. In the case of several pairs with the same number, we selected events at minimum distances. For tomography, we used pairs of events with a number of common phases less than or equal to 6. For the pair (1) –(2), we selected 524 paired events with a number of common phases of 2,690 and 1,436 for the P and S waves, respectively. In this case, 11 stations were involved. For the pair (2) –(3), the number of events was 1,325, and the number of the P and S picks were 7,076 and 4,900, respectively. The distribution of events together with the P and S ray paths for these cases are shown in Figure S1.

The tomography procedure is based on the modified version of the LOTOS code[29]. All the controlling parameters, such as grid spacing, smoothing and amplitude damping, were identical for all models. The most important parameters are presented in Table S2.

The workflow starts with the location using the one-dimensional (1D) velocity model. During the preliminary stage, the source coordinates are determined using the grid search method. To accelerate the calculations, the travel times during this stage were computed based

on a straight line approximation. Note that prior to data selection, all events were relocated using this algorithm.

The recurrent tomographic procedure starts with another stage of source locations using the bending algorithm for ray tracing[30] that uses the starting 1D model during the 1st iteration and updated three-dimensional (3D) velocity models for the following iterations.

The 3D distributions of the P and S velocity anomalies were parameterized using a set of nodes distributed in the study volume according to the ray path coverage. The parameterization grid was constructed during the first iteration and remained unchanged during the following iterations. The parameterization nodes were installed according to the distribution of rays. No nodes were installed in the ray density was less than 0.1 of the average value. In map view, the nodes were set regularly with the spacing of 2 km. In the vertical direction, the distance between nodes was inversely proportional to the ray density, but was not smaller than 1 km (see Table S2). To make the solution grid independent, we performed inversions in several grids having different basic orientations and then averaged the derived results. A special feature of the repeated tomography is that the grid files are created for one dataset and then are copied to the folder of another dataset.

The sensitivity matrix was computed along the ray paths derived after the source location step and then inverted using the LSQR method[31,32]. To stabilize the solution, we applied regularization by damping the amplitude and variations of the anomalies between nodes. The optimal values of the amplitude and flattening parameters were estimated based on the results of the synthetic modeling. The values of the regularization parameters, as well as the weights for computation of station and source corrections, are presented in Table S2. Note that these parameters were identical for all models presented here. Note that in this study, we used much

less data than that used in Koulakov et al.[16] which determined the crustal structure beneath the same volcano using the data of the temporary network. Therefore, here we used more conservative damping values than those in the previous study which was important in obtaining stable temporary variations.

After computing anomalies in all grids, we combined them using a regular mesh and then used the 3D model as a basic distribution for the next iteration. In total, we used five iterations. In the case of the time period 2002–2004, the absolute deviations of the residuals in the L1 norm were reduced for the P- and S-wave data from 0.13 s and 0.18 s to 0.079 s and 0.087 s (41% and 52%), respectively. During other periods, the variance reduction was similar or greater (see Table S3).

All the results presented in the paper can be reproduced using the data files and the program codes available at http://www.ivan-art.com/science/LOTOS/repeatomo.zip. This compressed file includes a Read_Me.pdf file with detailed guidelines on how to perform the calculations.

<u>Inversion of experimental data</u>

Based on the experimental data, we computed a total of four models, two for each series. In all cases, we performed five iterations and used the same inversion parameters. The distributions of the *Vp* and *Vs* anomalies and their differences are shown in one horizontal and one vertical section in Figures S2 and S3, respectively. In these sections, we show also the distributions of the relocated seismicity. The *Vp/Vs* ratio, computed by the division of the resulting P- and S-wave velocities, seems to be a very important parameter for exploring volcanoes, as it is particularly sensitive to the presence of liquid and gas phases. Therefore, we

show the distribution of this parameter in the main paper in Figures 3 and 4. Note that beneath the summit area, we observed a high *Vp* and a low *Vs*, which results in a high *Vp/Vs* ratio. This is a typical feature of many volcanoes[1,18] and is interpreted as a magma reservoir with a high content of liquid fluids and melts.

During both time periods, the changes in the *P* and *S* anomalies and *Vp/Vs* ratios exceeded 5% in some areas. These velocity changes were much stronger than the variations derived during the synthetic tests when identical models were recovered using data from different datasets (see next section). This result shows that the derived variations in both pairs of data subsets do actually represent changes in the physical properties of the Earth and were not caused by changes in ray configurations. Similar and even stronger temporal variations of Vp, Vs and Vp/Vs ratio were previously in other volcanoes during unrest periods. For example, for the case of another Alaskan volcano, Mt. Redoubt, in 2009, during a strong magmatic eruption, the dVs inside the volcano edifice changed the sign from strongly positive to strongly negative values[1]. This was explained by fast migration of fluids during the eruption. Similar changes as in the present study were detected for Nevado del Ruiz in Colombia[23].

Synthetic modeling

Synthetic modeling is an important stage of any tomographic study that allows for assessing the spatial resolution of the derived models and evaluation of optimal values of controlling parameters. In the case of repeated tomography, synthetic modeling is a necessary step to distinguish the actual variations in seismic velocities from artifacts related to changes in data distributions.

The workflow in synthetic modeling should represent as near as possible the case of experimental data analysis. The distributions of source-receiver pairs are identical to those used in the experimental case. A synthetic model is defined using a series of polygons and it is absolutely independent of the parameterization grid used for inversion. The synthetic travel times are computed using 3D ray tracing based on the bending method. The derived travel times were perturbed with random noise (0.02 and 0.05 s in our case) providing the same variance reduction as in the case of the experimental data analysis. In addition, the origin times and coordinates of the sources are randomly biased. The recovery procedure repeats the same steps of experimental workflow including the step of preliminary source locations.

Figure S4 presents the results for the checkerboard test, in which the synthetic model was defined as a series of alternating square anomalies o 8 × 8 km in size. In all cases, the P- and S-wave velocity anomalies had different signs to ensure strong variations in the Vp/Vs ratio. Figure S4 shows the resulting Vp/Vs ratio for two pairs of time intervals. When performing modeling using this test, we considered both cases of identical and variable models. Rows 1 and 3 represent cases of identical models with the amplitudes of the Vp and Vs anomalies equal to ±8%. In both series, the recovery results are very similar, and the differences between the resulting models are nearly zero. In the second case as shown in rows 2 and 4, the amplitudes of the anomalies in the first data subset remained ±8%, and in the second subset, they increased to ±9%. Although the resulting distribution of the Vp/Vs ratio appears similar, their difference clearly reveals variations representing the difference in the initial model.

Figure S5 shows the synthetic test with realistic anomalies representing the general patterns observed in the case of the experimental data inversion. Beneath the summit area, we defined a columnar anomaly with contrasted high Vp/Vs anomaly. As in the previous series of

tests, we started with recovering identical models (1 and 3 rows). As in the previous case, the recovered distributions of the Vp/Vs ratio appear identical, and their difference is near zero. Then, for the first series corresponding to 1996–2001 and 2002–2004, we defined two anomalies with the same amplitudes of the Vp and Vs anomalies (±7%) but of different shapes. For the period 2002–2004, the upper limit of the anomaly was ~2 km shallower than in the case of 1996–2001. Similarly, as was observed in the case of experimental data inversion, in the recovered models, one can clearly see the difference in the shape of the anomaly. In the difference plot (2nd row, 3rd column), one can see strong variations representing the ascent of the model.

For the second pair of periods, the shapes of the anomalies were identical, but the amplitudes were ±7% and ±5% for the periods 2002–2004 and 2005–2012, respectively. The inversion results for these two cases provide similar shapes of the recovered anomalies but show clear variations in the difference plot (4th row and 3rd column). All these tests show that the scenario identified from the analysis of the experimental data inversion results appears to be robustly resolvable and realistic.

References for supplementary information:

29. Koulakov, I., LOTOS code for local earthquake tomographic inversion. Benchmarks for testing tomographic algorithms. *Bulletin of the Seismological Society of America* **99**, 194–214 (2009).
30. Um, J. & Thurber, C.H. A fast algorithm for two-point seismic ray tracing. *Bulletin of the Seismological Society of America* **77**, 972-986 (1987).

31. Paige, C.C. & Saunders, M.A. LSQR: An Algorithm for Sparse Linear Equations and Sparse Least Squares. *ACM Transactions on Mathematical Software (TOMS)*, **8**, 43–71 (1982).

32. Nolet, G. Seismic wave propagation and seismic tomography. *Seismic Tomography*, edited by G. Nolet, 1–23, Reidel, Dordrecht (1987)

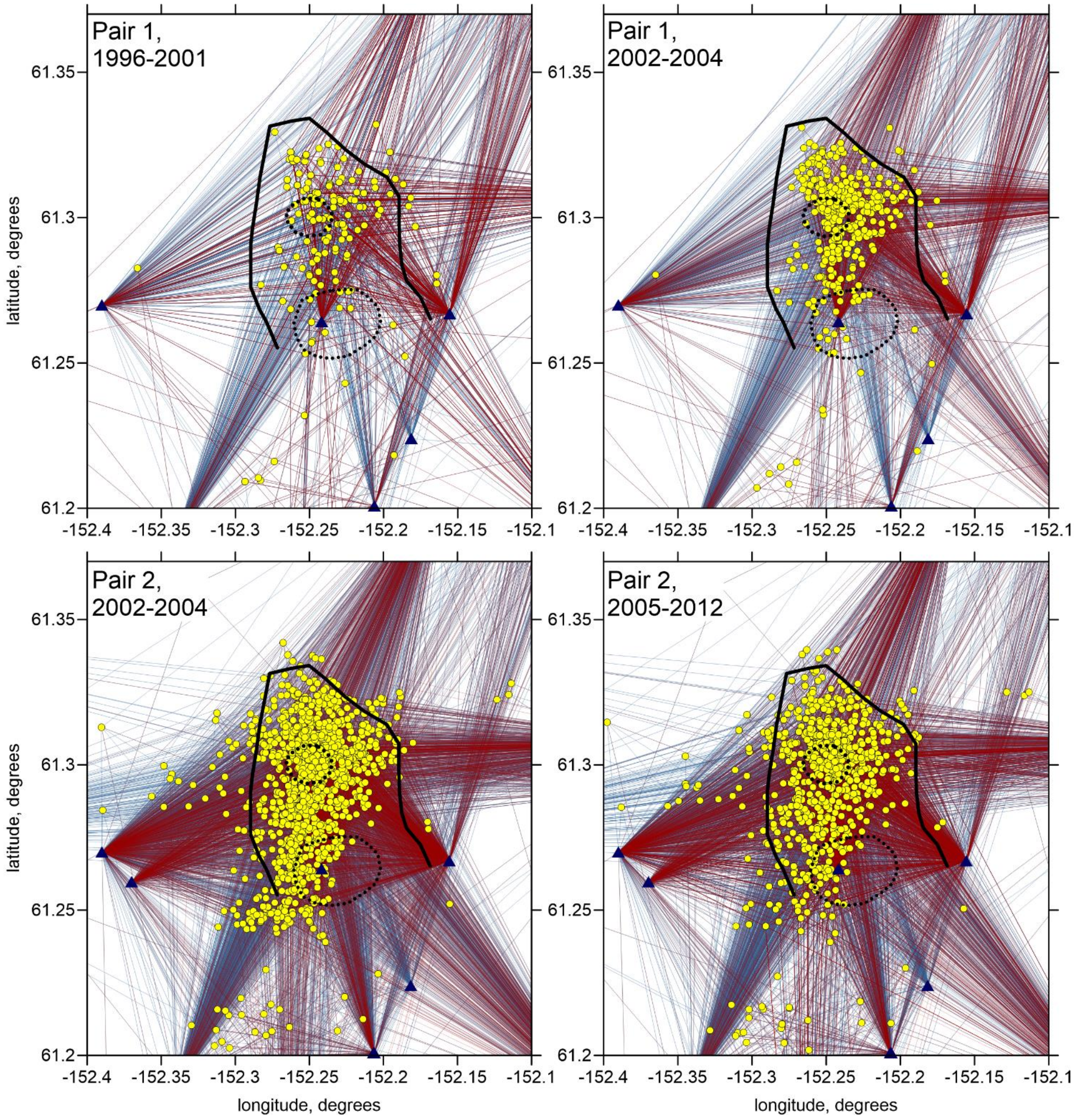

Figure S1. Selections of similar datasets for two pairs of time periods. Yellow dots depict events; blue and red lines are the ray paths for the P and S waves, respectively. The lack solid line depicts the limits of the caldera; the dotted lines indicate the Mt. Spurr summit and Crater Peak.

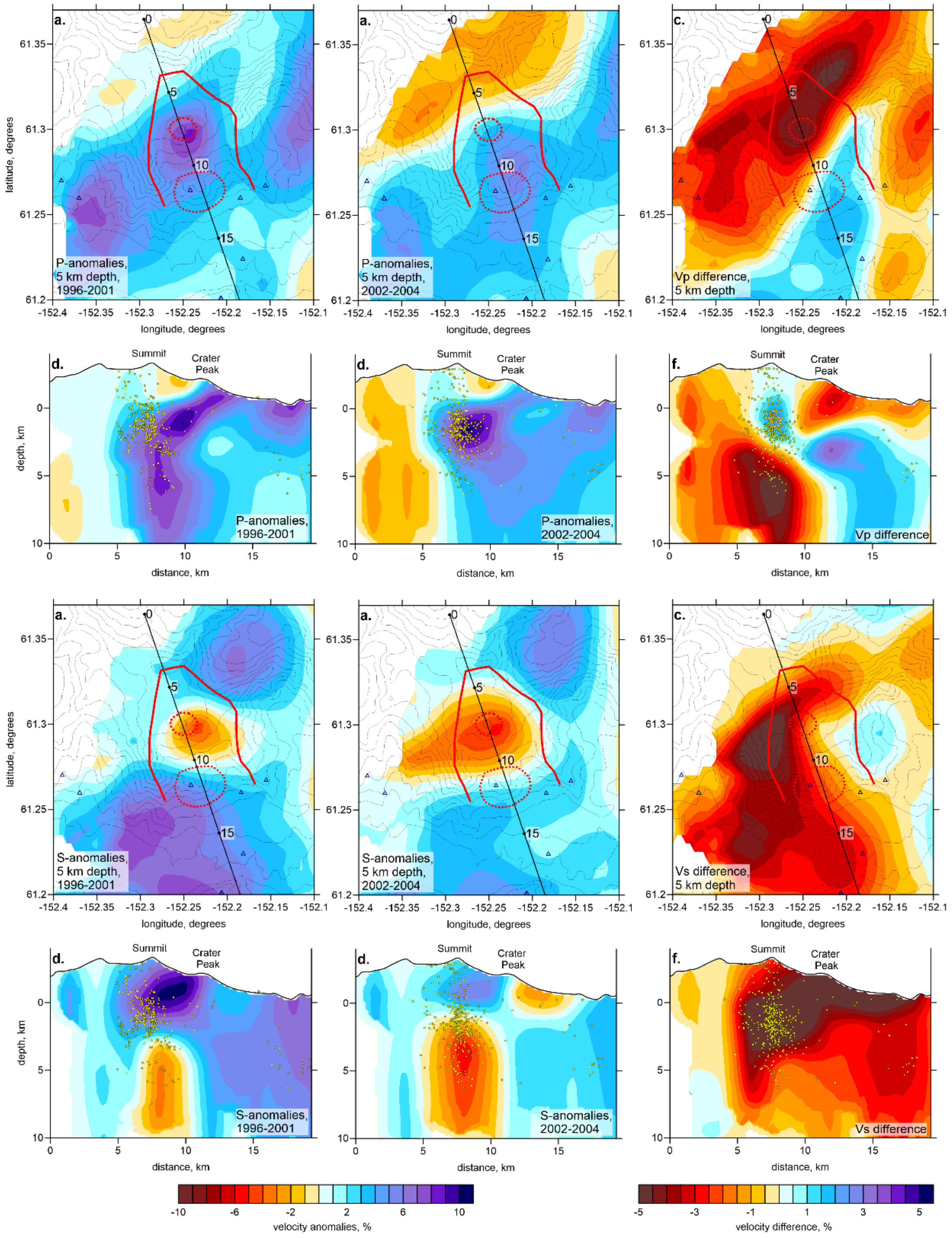

Figure S2. Distribution of the P- and S-wave velocity anomalies derived from the inversion of the datasets from the two time periods: 1996–2001 and 2002–2004. The results are plotted at 5 km depth (upper row) and in the vertical section (lower row). The location of the section is indicated in the maps. The right column presents the difference between velocities in the corresponding horizontal and vertical sections. The yellow dots in the vertical sections depict the events within a distance of 5 km from the profile. The red solid line depicts the limits of the caldera; the red dotted lines indicate the Mt. Spurr summit and Crater Peak.

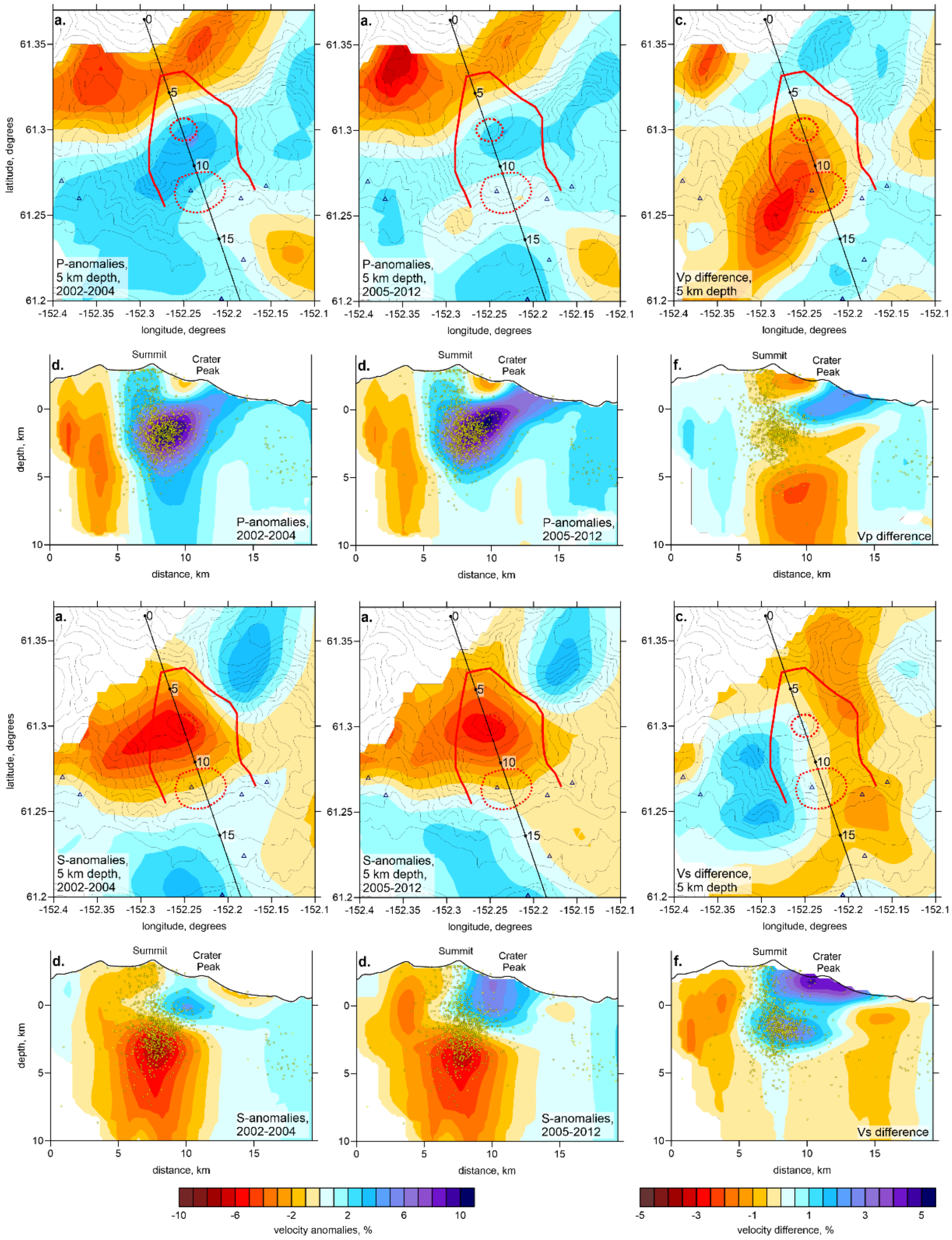

Figure S3. Same as Figure S2, but for another pair of time episodes: 2002–2004 and 2005–2012.

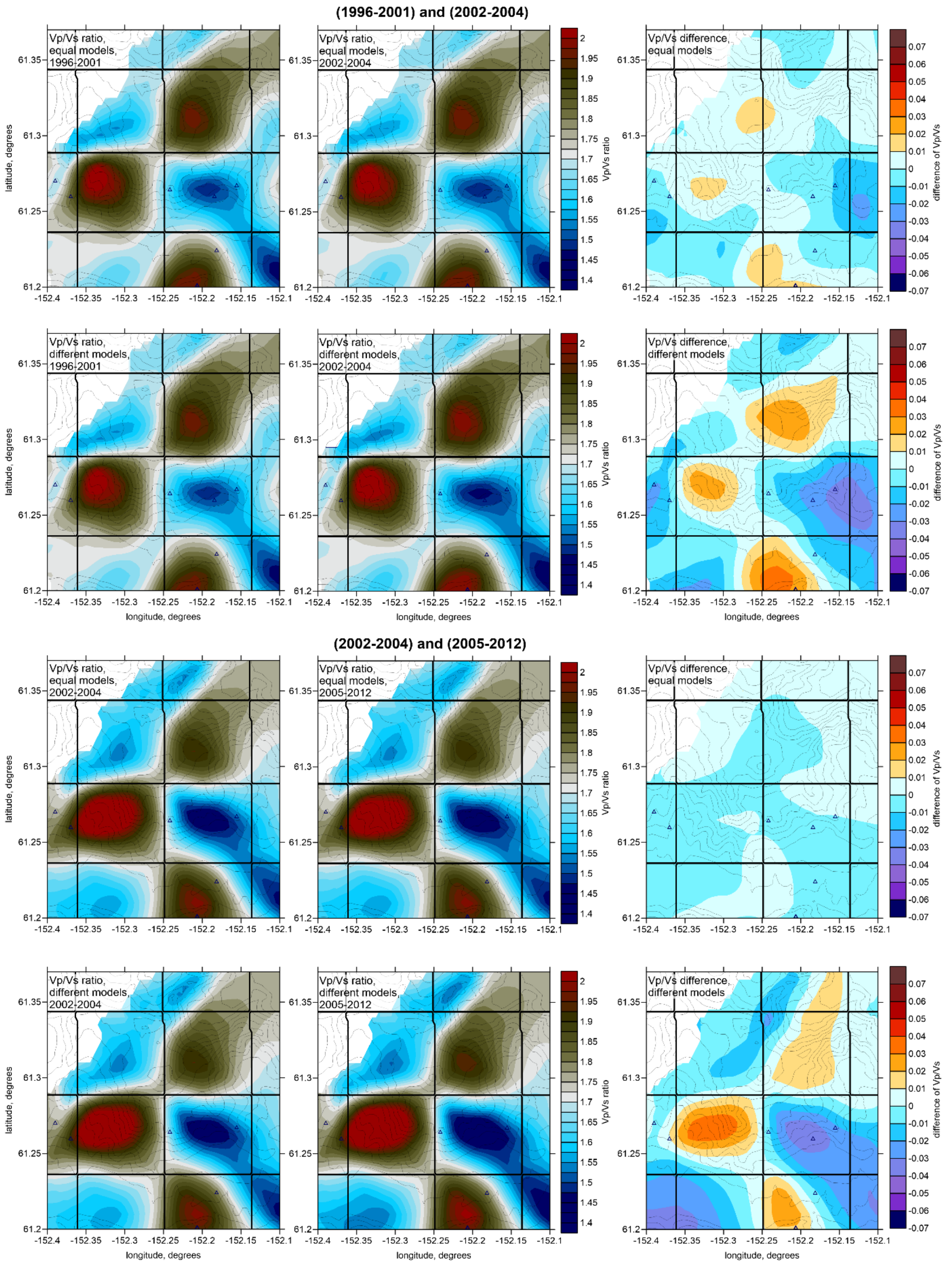

Figure S4. Checkerboard tests to check the ability of the recovering anomalies for cases with identical and different amplitudes. The solid line depicts the shape of the initial anomalies. The 1st and 2nd rows correspond to the datasets of 1996–2001 and 2002–2004, and the 3rd and 4th rows for 2002–2004 and 2005–2012, respectively. The 1st and 2nd columns show the results for the Vp/Vs ratio for the different time periods; the right column is the difference. The 1st and 3rd rows show the results for identical synthetic models (±8% in all cases). The 2nd and 4th rows show the results for checkerboards having different amplitudes (±8% and ±9% in the 1st and 2nd columns, respectively).

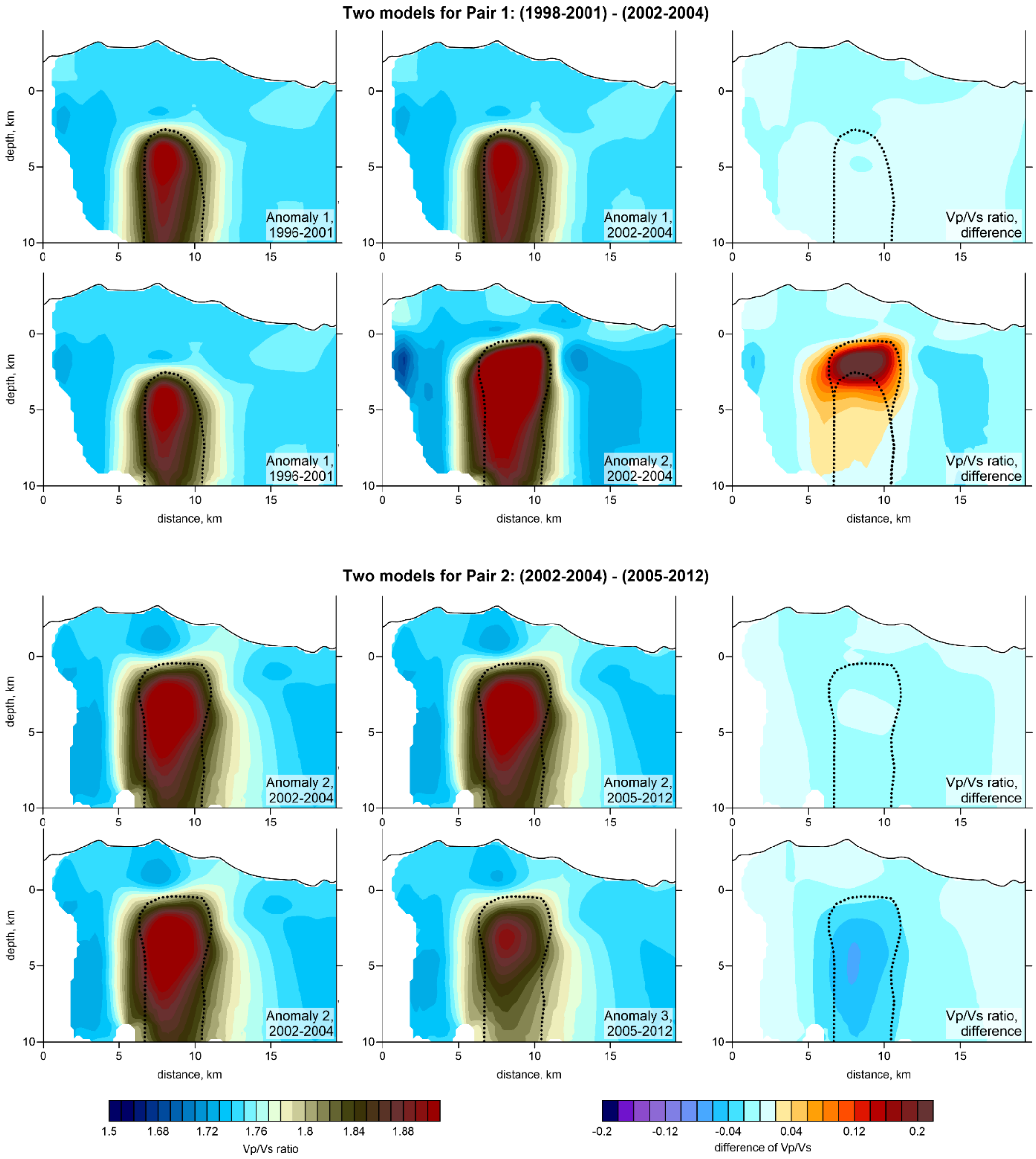

Figure S5. Synthetic modeling with anomalies representing the realistic magma reservoir. The dotted lines depict the shapes of the initial anomalies. The 1st and 2nd rows correspond to the datasets of 1996–2001 and 2002–2004, and the 3rd and 4th rows are for 2002–2004 and 2005–2012, respectively. The 1st and 2nd columns show the results for the Vp/Vs ratio for different time periods; the right column is the difference. We considered three types of anomalies: anomaly 1 is deeper than anomaly 2 and has the same amplitude of the P- and S-wave velocity anomalies (±7%). Anomalies 2 and 3 have identical shapes, but different amplitudes (±7% and ±5%, respectively).

Table S1. Characteristics of seismic stations on the Spurr volcano used in this study

Station name, (components)	Latitude, degrees	Longitude, degrees	Elevation, m	Instrument type	Open (closing) date
BGL (1C)	61.2663	-152.3913	1,127	L4	1989/08/13 -
BKG (1C)	61.0696	-152.2650	1,009	L4	1991/07/01 –
CGL (1C)	61.3071	-152.0090	1,082	L4	1981/09/22 –
CKL (1C)	61.1958	-152.3400	1,281	L4	1989/08/05 –
CKN (1C)	61.2234	-152.1838	735	L4	1991/09/19 –
CKT (1C)	61.2002	-152.2085	975	L4	1992/09/16 –
CP2 (1C)	61.2636	-152.2441	1,981	L4	1992/10/23 –
CRP (3C)	61.2664	-152.1578	1,622	L4 (3C)	1981/08/26 –
NCG (1C)	61.4031	-152.1590	1,244	L4	1989/08/06 –
SPBG (3C)	61.2591	-152.3722	1,087	CMG-6TD	2004/09/09 –
SPCG (3C)	61.2913	-152.0228	1,329	CMG-6TD	2004/09/08 –
SPCN (3C)	61.2244	-152.1854	735	CMG-6TD	2010/09/01 –
SPCP (3C)	61.2655	-152.1550	1,616	CMG-6TD	2010/10/02 –
SPCR (3C)	61.2003	-152.2091	984	CMG-6TD	2004/09/08 –
SPNN (3C)	61.3662	-152.7012	1,666	CMG-6TD	2011/08/01 –
SPNW (1C)	61.3471	-152.6039	1,040	L4	2004/08/17 – 2011/08/01
SPU (1C)	61.1811	-152.0566	800	L4	1971/08/10 –
SPWE (1C)	61.2728	-152.5614	1,327	L4	2004/08/18 -

Table S2. Values of the main controlling parameters used for inversion, which were identical for all models based on experimental and synthetic data.

Description of parameters	Values
Total number of iterations	5
Minimum number of picks per event	6
Maximum residual (s)	0.5
Horizontal grid spacing (km)	2
Minimum vertical grid spacing (km)	1
Orientations of the parameterization grids (degrees)	0, 22, 45 and 66
Amplitude damping for the P and S wave velocity models	0.3 and 0.6
Smoothing for the P and S wave velocity models	0.6 and 1.2
Station correction weight in inversion	1
Source correction weights for the horizontal and vertical coordinates and origin times	5, 5 and 5

Table S3. Information regarding numbers of data and values of residuals for two series of repeated tomographic inversions

Series	Time intervals	Number of events	Data type:	Number of rays	Starting residual, s	Final residual, s	Residual reduction, %
Series 1	1996–2001	524	P-data:	2690	0.1263	0.0547	56.65
			S-data	1436	0.1833	0.0583	68.17
	2002–2004	524	P-data:	2690	0.1247	0.0639	48.74
			S-data	1436	0.1834	0.0670	63.44
Series 2	2002–2004	1325	P-data:	7076	0.1340	0.0790	41.02
			S-data	4900	0.1834	0.0872	52.41
	2005–2012	1325	P-data:	7076	0.1335	0.0738	44.67
			S-data	4900	0.1860	0.0805	56.70
